# The MarR-Type Regulator PA3458 Is Involved in Osmoadaptation Control in *Pseudomonas aeruginosa*

**DOI:** 10.3390/ijms22083982

**Published:** 2021-04-12

**Authors:** Karolina Kotecka, Adam Kawalek, Kamil Kobylecki, Aneta Agnieszka Bartosik

**Affiliations:** Institute of Biochemistry and Biophysics, Polish Academy of Sciences, 02-106 Warsaw, Poland; ka.kotecka@ibb.waw.pl (K.K.); a.kawalek@ibb.waw.pl (A.K.); kkobylecki@ibb.waw.pl (K.K.)

**Keywords:** *Pseudomonas aeruginosa*, *PA3458*, MarR-type transcriptional regulator, repressor, osmoadaptation

## Abstract

*Pseudomonas aeruginosa* is a facultative human pathogen, causing acute and chronic infections that are especially dangerous for immunocompromised patients. The eradication of *P. aeruginosa* is difficult due to its intrinsic antibiotic resistance mechanisms, high adaptability, and genetic plasticity. The bacterium possesses multilevel regulatory systems engaging a huge repertoire of transcriptional regulators (TRs). Among these, the MarR family encompasses a number of proteins, mainly acting as repressors, which are involved in response to various environmental signals. In this work, we aimed to decipher the role of PA3458, a putative MarR-type TR from *P*. *aeruginosa*. Transcriptional profiling of *P. aeruginosa* PAO1161 overexpressing *PA3458* showed changes in the mRNA level of 133 genes; among them, 100 were down-regulated, suggesting the repressor function of PA3458. Concomitantly, ChIP-seq analysis identified more than 300 PA3458 binding sites in *P. aeruginosa*. The PA3458 regulon encompasses genes involved in stress response, including the *PA3459–PA3461* operon, which is divergent to *PA3458*. This operon encodes an asparagine synthase, a GNAT-family acetyltransferase, and a glutamyl aminopeptidase engaged in the production of N-acetylglutaminylglutamine amide (NAGGN), which is a potent bacterial osmoprotectant. We showed that PA3458-mediated control of *PA3459*–*PA3461* expression is required for the adaptation of *P*. *aeruginosa* growth in high osmolarity. Overall, our data indicate that PA3458 plays a role in osmoadaptation control in *P. aeruginosa*.

## 1. Introduction

*P. aeruginosa* is a common bacterium that survives in variable environmental conditions, including the human body, and it is the main agent causing nosocomial infections of the lungs, wounds, blood, and urinary tracts [1]. *P. aeruginosa* is the leading cause of mortality in cystic fibrosis patients [2]. Treatment of the acute and chronic infections caused by this bacterium is very difficult due to its adaptability and intrinsic antibiotic resistance mechanisms [3]. *P. aeruginosa* has complex regulatory systems, which allow it to combat environmental stressors with the use of different mechanisms; e.g., in the presence of an antibiotic, cells could use efflux pumps [4] or form biofilm [5]. The multilevel regulatory network allows *P. aeruginosa* to modulate the metabolism to endure different stress conditions e.g., heat shock [6], the presence of antimicrobials [7], photooxidative [8], or osmotic stress [9,10].

Quick and adequate responses of *P. aeruginosa* to different environmental signals are possible due to complex regulatory systems, which engage more than 500 characterized or potential transcriptional regulators and two-component regulatory system proteins [11], comprising almost 10% of all its genes. Nineteen prokaryotic transcriptional regulator families have been evaluated so far [12], including the MarR family of transcriptional regulators.

Acting mainly as repressors, MarR-type TRs are involved in the control of different processes e.g., adhesion, virulence, and antibiotic resistance [13]. The first and the best-studied regulator from this family is the multiple antibiotic resistance regulator MarR from *Escherichia coli* [14]. It was originally identified as a component encoded within the *E. coli marRAB* locus, which negatively regulated the expression of this operon [15]. The MarR works as a dimer, and the N-terminal part of the protein is important for protein oligomerisation. The C-terminal part of MarR contains the winged-helix-turn-helix (wHTH) motif, which is involved in DNA recognition and binding [14]. The MarR binding to DNA is inhibited by salicylate, forming a complex with MarR [16]. MarA acts as an autoactivator of *marRAB* but is also involved in the regulation of multiple other genes affecting resistance to antibiotics and other environmental hazards [17,18].

Representatives of the MarR family often show similar organization of genetic loci with a regulator gene and usually divergently oriented target genes. The regulator controls the activity of its gene and a divergently oriented gene or operon by binding to the intergenic region and blocking RNA polymerase by steric inhibition [19].

A vast number of the described MarR-type TRs are involved in the control of virulence and stress response genes, e.g., HpaR of *Xanthomonas campestris* [20]; PrhN of *Ralstonia solanacearum* [21]; SlyA of *Dickeya dadantii* [22], *Enterococcus faecalis* [23,24], or *Salmonella typhimurium* [25]; YodB of *Bacillus subtilis* [26]; CosR in *Corynebacterium glutamicum* [27] or BmoR of *Bacteroides fragilis* [28].

In *P. aeruginosa* PAO1, thirteen genes coding for MarR-type regulators were identified based on NCBI [29] and pseudomonas.com [30] databases. The best-characterized known and important from the clinical point of view is MexR (PA0424), which negatively regulates multidrug efflux systems [31]. Mutation in the *mexR* gene causes higher resistance to antibiotics [32]. It was shown that the oxidation stress may serve as a signal to MexR, causing inhibition of binding to DNA [33]. Similarly, the *P. aeruginosa ohrR* (*PA2849*) and *ospR* (*PA2825*) both encoded MarR-type regulators, which control hydroperoxide stress response [34]. Another MarR-type TR from *P*. *aeruginosa*, HudR (PA0253), acts as a repressor of the *hud*A gene encoding a virulence-attenuating factor [35].

The PA3458 protein from *P. aeruginosa* has been classified in silico as a putative MarR-type transcriptional regulator. The *PA3458* gene is part of the *parA/parB* regulatory network (it is up-regulated in *P. aeruginosa parA* and *parB* mutants) [36]. There are more than 690 and 1160 genes in ParA and ParB regulon, respectively. ParA and ParB are involved in chromosome segregation, which is manifested by anucleate cell formation and slight cessation of mutants growth [37,38], but genes in their regulon represented different functional categories, among them a huge amount of genes encoding transcriptional regulators [36]. Their role in *P. aeruginosa* biology and metabolism in many cases awaits elucidation.

The aim of this study was identification of the function of the MarR-family transcriptional regulator PA3458 in *P. aeruginosa.*

## 2. Results

### 2.1. The PA3458–PA3461 Gene Cluster of P. aeruginosa

**Figure 1:**
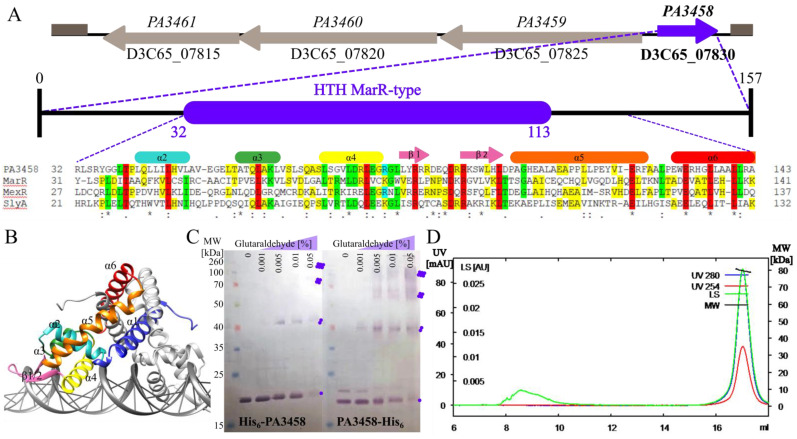
Characterization of PA3458 protein from *P. aeruginosa*. (**A**) Genomic context of the *PA3458* gene in the PAO1161 genome and the domain structure of the PA3458 protein. The gene IDs from PAO1 and PAO1161 strains are presented. Alignment represents comparison of PA3458 with corresponding regions of MarR (GenBank: AAK21292.1), MexR (GenBank: AVV61365.1), and SlyA (GenBank: AAL55673.1). Sequences were aligned using the Clustal Omega [39] with identical residues in all proteins marked with red, in three sequences with green or in two with yellow, respectively. The secondary structure elements are marked with colored boxes corresponding to predicted domains presented in (**B**). (**B**) Structural model of PA3458 dimer bound with DNA. The model was built using SWISS-MODEL and HDOCK [40,41]. (**C**) Oligomerization state of purified His_6_-PA3458 and PA3458-His_6_ assayed by crosslinking of purified protein with glutaraldehyde. Samples were separated using SDS-PAGE and immunodetected with anty-His antibodies. A violet dot indicates a monomer, two dots indicate a dimer, four indicate tetramers, and six indicate hexamers. (**D**) Size exclusion chromatography (SEC) with multi-angle static light scattering (MALS) analysis for PA3458-His_6_. Left axis—UV and light scattering (LS) absorption, right axis—molecular weight of protein (MW).

The *PA3458* gene in *P. aeruginosa* PAO1161, similarly to PAO1, is encoded in the opposite transcriptional orientation to the predicted *PA3459*–*PA3461* operon (Figure 1A), which according to the pseudomonas.com database encodes an asparagine synthase (PA3459), a GNAT-family acetyltransferase (PA3460), and a glutamyl aminopeptidase (PA3461). Comparative microarray analysis showed that the *PA3459*–*PA3461* genes were up-regulated in *P. aeruginosa* cells in response to osmotic stress [9]. They encode proteins involved in the production of an N-acetylglutaminylglutamine amide (NAGGN), a potent osmoprotectant conferring bacterial cells with resistance to osmotic stress [9]. The close genomic localization of the *PA3458* gene to the *PA3459*–*PA3461* gene cluster suggests a functional relationship.

The *PA3458* gene encodes a protein classified in silico as a potential MarR-type transcriptional regulator. It encodes a small protein of 157 residues (17.6 kDa), with a predicted winged helix-turn-helix (wHTH) domain responsible for DNA binding. Upon comparison of the part of the protein encompassing the wHTH motif with DNA binding domains of MarR-type representatives, MarR from *E. coli* [42], MexR from *P. aeruginosa* [31], and SlyA from *Enterobacteriaceae* [43] showed the presence of highly conserved residues potentially engaged in DNA binding (Figure 1A). Secondary structure prediction using SWISS-MODEL and HDOCK servers [40,41] predicted a dimer of a triangular shape formed by two PA3458 monomers, each consisting of six α-helices and two β-strands arranged in the order α1-α2-α3-α4-β1-β2-α5-α6 in the primary structure (Figure 1A,B). The dimerization interface is formed by helices α1, α5, and α6 of two monomers, while helices α3 and α4 form a helix-turn-helix (HTH) involved in DNA binding. Between helices α4 and α5 is a wing motif comprising two antiparallel β-strands and their connecting loop. The wHTH with α-helix 4 (α4) is critical for interactions with DNA (Figure 1B). The predicted structure of PA3458 resembles the structures of other MarR family proteins [44,45].

To check the oligomeric state of PA3458, two methods were used. Glutaraldehyde crosslinking of purified His_6_-PA3458 and PA3458-His_6_, followed by Western blot analysis, showed the presence of dimmers as well as higher-order complexes corresponding in size to tetramers (Figure 1C). In the case of PA3458-His_6_ fusion, the oligomers were crosslinked more efficiently, which suggests that the free N-terminus of the protein may be required for PA3458 self-assembly. Similarly, analysis of the oligomeric state of PA3458-His_6_ using SEC-MALS demonstrated that this protein existed preferentially as a tetramer in solution under the tested conditions (Figure 1D). These data indicates that PA3458 is able to self-assemble and creates oligomers. The oligomeric state of the protein may play an important regulatory role in protein action as the transcriptional regulator.

### 2.2. Effect of PA3458 Lack or Excess on Bacterial Growth

**Figure A1:**
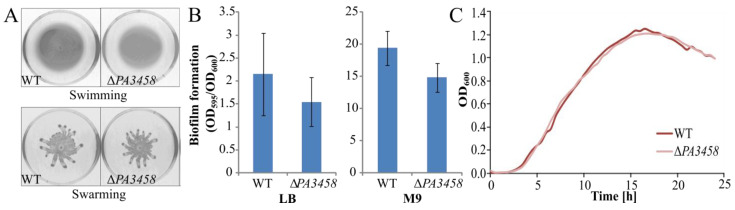
Phenotypic characterisation of PAO1161 Δ*PA3458* deletion mutant. (**A**) Growth of PAO1161 and Δ*PA3458* mutant on swimming and swarming medium. (**B**) Biofilm formation of PAO1161 *leu*^−^ and Δ*PA3458* mutant on LB or minimal medium with citrate. Data represent mean from five independent replicates. (**C**) Growth of PAO1161 and Δ*PA3458* mutant on LB.

To assign a biological function to PA3458, the chromosomal mutant of PAO1161 Δ*PA3458* was constructed using the allele exchange method (Table A1 and Table A2). The phenotype analysis of the Δ*PA3458* mutant in comparison with WT did not show significant changes in bacterial growth, motility abilities, or biofilm formation under tested conditions (Figure A1).

**Figure 2:**
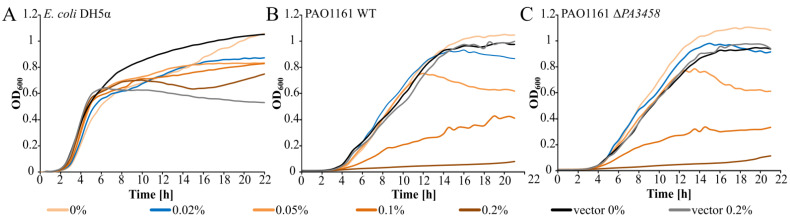
Effect of PA3458 excess on bacterial growth. *E. coli* DH5α (**A**) or *P. aeruginosa* PAO1161 (**B**) or Δ*PA3458* (**C**) mutant strains carrying empty vector pKGB8 a*raBAD*p or pKKB2.11 *araBADp-PA3458* were grown in Luria–Bertani (LB) broth under selection with the indicated concentration of inducer arabinose (0 to 0.2%). The blue line indicates the growth in the presence of 0.02% arabinose, conditions selected for RNA-seq analysis. Data represent mean optical density at 600 nm (OD_600_) from three independent replicates. Standard deviations are less than 20% and are not shown for clarity.

To further determine the influence of PA3458 on bacterial growth, the *PA3458* gene was cloned under the control of *araC*-*BAD*p in the broad host range expression vector pKGB8, which is the derivative of pBBR1-MCS1 [46,47]. The growth of *E. coli* DH5α (pKKB2.11 *araBAD*p*-PA3458*) and DH5α (pKGB8 *araBAD*p) cells conducted under selection in Luria–Bertani (LB) broth in the presence of different concentrations (0–0.2%) of inducer arabinose was tested. Irrespective of the *PA3458* overexpression or its lack, no difference in the kinetics of bacterial growth was detected under tested conditions (Figure 2A).

The growth of *P. aeruginosa* PAO1161 and PAO1161 Δ*PA3458* mutant with pKKB2.11 *araBAD*p*-PA3458* or empty vector (pKGB8 *araBAD*p*)* in the presence of different concentrations of arabinose was also tested. The slight PA3458 overproduction in the presence of 0.01–0.05% arabinose did not affect the bacterial growth visibly (Figure 2B,C). Induction of the *PA3458* expression by the addition of the higher concentrations of arabinose (0.1–0.2%) to the cultures affected significantly kinetics of bacterial growth, with the strongest slow down effect observed for the highest arabinose concentration tested (0.2%). An addition of arabinose did not affect the growth of cells carrying the empty vector.

These data show that the higher level of PA3458 in the cells has a strong negative impact on the growth of *P. aeruginosa* but not of *E. coli*, suggesting the existence of the sensitive targets of PA3458 action in *P. aeruginosa* cells.

### 2.3. Effect of Increased PA3458 Level on Gene Expression

To identify the potential functions and pathways associated with the action of PA3458 in *P. aeruginosa*, we set out to determine its regulon. The impact of slight PA3458 overproduction on gene expression was analyzed using RNA-seq analysis, and concomitantly, PA3458 binding sites in the *P*. *aeruginosa* genome were identified using chromatin immunoprecipitation-sequencing (ChIP-seq). RNA was isolated from logarithmically growing (OD_600_ 0.5) PAO1161 (pKKB2.11 *arapBAD-PA3458*) cells grown under selection in LB with 0.02% arabinose representing conditions of the slight PA3458 overproduction, not perturbing bacterial growth (hereafter called PA3458+) and PAO1161 (pKGB8 *araBAD*p) cultures grown under selection in LB with 0.02% arabinose (empty vector control, hereafter called EV+). We used the low concentration of arabinose (0.02%) to induce the PA3458 production and not affect the cell growth, which is observed when higher concentrations of arabinose are used, leading to high PA3458 overproduction and strong inhibition of bacterial growth (Figure 2B).

**Figure 3:**
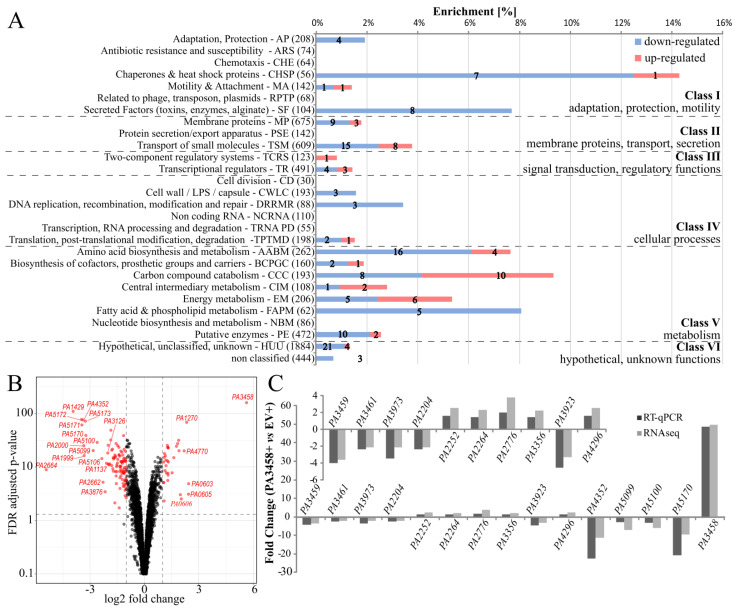
Identification of *P*. *aeruginosa* genes affected by PA3458. Comparative transcriptome analysis was performed for PA3458 overproducing cells PA3458+ vs. EV+ *P. aeruginosa* PAO1161 cells. (**A**) Enrichment of PseudoCAP functional categories [30] for 133 genes showing changes in mRNA level in response to mild PA3458 abundance (FC ≤ −2 or ≥ 2, FDR adjusted *p*-value ≤ 0.01). The numbers in brackets show the number of all genes in the PAO1 genome in the indicated PseudoCAP category. One gene could be classified into more than one category. Numbers in red or blue bars denote the number of up- or down-regulated genes, respectively, in each category. The PseudoCAP categories were grouped into six more general classes. (**B**) Volcano plot visualization of differential expression in analysis between transcriptomes of PA3458+ vs. EV+ cells. Each point in the volcano plot represents one gene, and the dashed lines represent the cut-off values used. The red dots represent the most significant changes. (**C**) Validation of RNA-seq data by RT-qPCR analysis. The RT-qPCR was performed using RNA samples obtained for the same conditions as samples used for RNA-seq analysis. Data represent mean fold change for three biological replicates. The *proC* was used as a reference gene.

Comparative transcriptome analysis of PA3458+ vs. EV+ cells indicated 133 loci with an altered expression in response to the excess of PA3458 in the PAO1161 cells (fold change (FC) ≤ −2 or ≥ 2, false discovery rate (FDR) adjusted *p*-value ≤ 0.01) (Figure 3A; Table S1). Among identified loci, 33 exhibited increased mRNA levels and 100 showed decreased mRNA levels. Genes with altered expression mostly belong to the transport of small molecules and amino acid metabolism PseudoCAP categories [30], 23 and 20 genes, respectively. Interestingly, most of these genes were down-regulated. The category with the highest enrichment (14%) encompasses eight genes (seven down-regulated) encoding chaperones and heat shock proteins (Figure 3A). Almost all genes assigned to class I, encompassing genes with adaptation, protection, and motility functions were also down-regulated. Additionally, the down-regulated genes encompass all, except one, representatives encoding functions connected with cellular processes (class IV), as well as many genes assigned to class II and V; thus, they are engaged in membrane functions and metabolism (Figure 3A).

The volcano plot highlighted genes with the most significant changes (Figure 3B). For selected genes, RT-qPCR verification of changes observed in RNA-seq analysis was performed (Figure 3C). Importantly, for all tested genes, the direction of changes is consistent for both analyses.

### 2.4. Identification of PA3458 Binding Sites in P. aeruginosa

**Figure 4:**
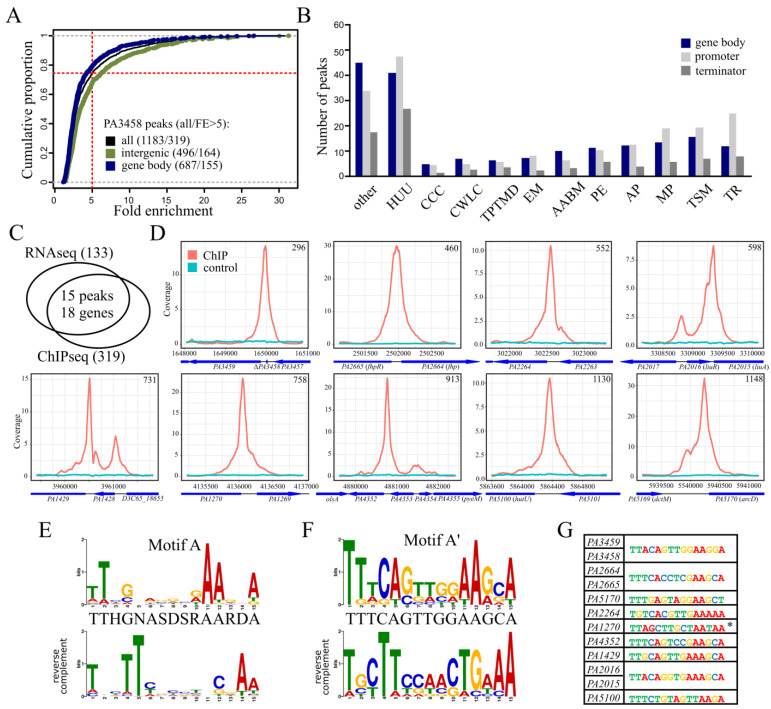
Identification of PA3458 binding sites in *P. aeruginosa*. Chromatin immunoprecipitation-sequencing (ChIP-seq) analysis was performed using PAO1161 strain expressing *PA3458-FLAG*. (**A**) Distribution of fold enrichment (FE) values for all detected peaks and separately for those identified in intergenic regions or gene body, respectively. FE cut-off value 5 is shown as the red dotted line. In panels B-G, analysis for 319 peaks with FE > 5 is presented. (**B**) PseudoCAP analysis of genes with PA3458 binding sites in a promoter, gene body, or terminator (both genes were included in the case of divergent promoters); HUU—hypothetical, unclassified, unknown; CCC—carbon compound catabolism; CWLC—cell wall/LPS/capsule; TPTMD—translation, post-translational modification, degradation; EM—energy metabolism; AABM—amino acid biosynthesis and metabolism; PE—putative enzymes; AP—adaptation, protection; MP—membrane proteins; TSM—transport of small molecules; TR—transcriptional regulators [30]. (**C**) Overlap between RNA-seq and ChIP-seq analyses. (**D**) ChIP-seq signal over regions encompassing selected PA3458 binding sites. The plots show coverage with reads for indicated positions in PAO1161 genome, normalized per genome coverage (RPGC) and averaged for ChIP replicates. Genes are presented as blue arrows; only names of PAO1 orthologs are shown for clarity. (**E**,**F**) The consensus sequence logos of predicted PA3458 binding sites, obtained by MEME-ChIP [49,50] using the zero or one occurrence per sequence option and sequences corresponding to 200 bp regions around summits of 319 peaks (FE > 5) (motif A, logo built based on 306 sequences) (**E**), as well as nine peak summits located in promoter regions of regulated genes (marked in Table S4) (motif A’, logo built from eight sequences, Table S3) (**F**). The height of an individual letter represents the relative frequency of the nucleotide at a particular position. The consensus sequence for each motif as well as reverse complement presentation of sequence logos are shown below. (**G**) Motif sites (motif A’ or motif A*) identified in promoter regions of regulated genes (statistic is presented in Table S3).

**Figure A2:**
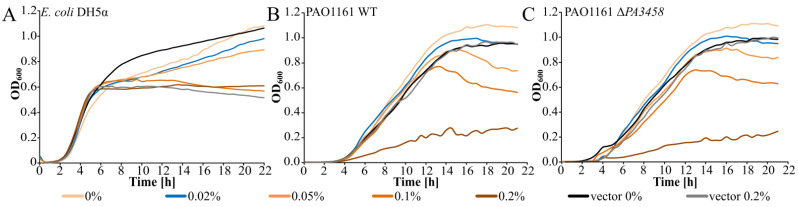
Effect of PA3458-FLAG overproduction on kinetics of growth. *E. coli* DH5α (**A**), or *P. aeruginosa* PAO1161 (**B**) or Δ*PA3458* (**C**) mutant strains carrying empty vector pABB28.3 a*raBADp-flag* or pKKB2.11 *araBADp-PA3458-flag* were grown in LB under selection with the indicated concentration of inducer arabinose (0 to 0.2%). The blue line indicates the growth in the presence of 0.02% arabinose, which are conditions selected for ChIP-seq analysis. Data represent mean OD_600_ from three independent replicates. Standard deviations are not shown for clarity.

To identify the PA3458 binding sites in the PAO1161 genome, the ChIP-seq analysis was conducted using the PAO1161 Δ*PA3458* strain carrying pKKB2.12 (*araBAD*p*-PA3458-flag*) and anti-FLAG antibodies. The PA3458-FLAG fusion protein, when overproduced, exhibited a similar effect on *P. aeruginosa* growth as the untagged PA3458 (Figure A2), confirming the functionality of the fusion protein. The ChIP-seq analysis was performed on DNA isolated from PAO1161 Δ*PA3458* (pKKB2.12 *araBAD*p*-PA3458-flag*) cells grown with 0.02% arabinose, conditions of the slight protein overproduction not perturbing bacterial growth (hereafter called PA3458-F+) and PAO1161 Δ*PA3458* (pABB28.3 *araBAD*p*-flag*) control cultures grown in the same conditions (hereafter called EV-F). The anti-FLAG immunoprecipitated DNA from three biological samples of PA3458-F+ and one EV-F was sequenced, and the reads were mapped to the *P. aeruginosa* PAO1161 genome [48]. To identify the sequences corresponding to the PA3458 binding sites, peak calling on merged data for three replicates was performed, as justified by the high similarity of coverage data between replicates (data not shown). Data obtained for EV-F sample were used to eliminate the regions enriched non-specifically during the ChIP procedure with the antibodies. Using the FDR-adjusted *p*-value cut-off value of 0.05 and fold enrichment (FE) > 1.5, 1183 ChIP-seq peaks were identified (Figure 4A; Table S2). The peaks displayed FE up to 31.28, with a median of 7.0. We have noted that the fold enrichment for peaks with intergenic summits was generally higher than for those that were located in gene bodies (Figure 4A). To decrease the number of non-specific binding sites, further analyses were limited to 319 peaks with FE > 5 (Table S3). Summits of 164 peaks from 319 mapped to intergenic regions (Figure 4A). A global analysis of functional categories of the genes ascribed to the PA3458-binding regions identified the transport of small molecules, membrane proteins, and transcriptional regulators as the most represented categories (Figure 4B).

Comparison of the ChIP-seq data with RNA-seq data (Figure 4C) pointed out four intragenic and 11 intergenic PA3458 binding sites in proximity of a gene with transcript level affected by PA3458 excess (Table 1). Of these, nine loci showed PA3458 bound sites upstream of genes regulated by PA3458 abundance, suggesting a direct involvement of PA3458 in their regulation (Figure 4D). Interestingly, they encode proteins potentially involved in stress response (PA2664, PA2665, PA1429, PA1270, PA4352, and PA3459) and amino acid metabolism (PA2264, PA5170, PA5100, and PA2015) (Table 1).

**Figure 5:**
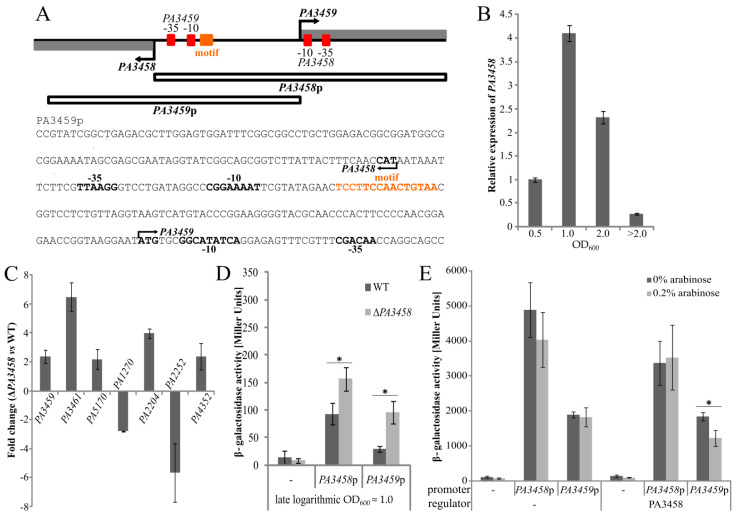
Direct regulation of gene expression by PA3458. (**A**) The scheme of the *PA3458/PA3459* promoter region used in the analysis. The sequence of *PA3459*p cloned upstream of a promoter-less *lacZ* reporter gene in pCM132 with marked PA3458 binding motif and predicted -10, -35 promoter sequences are presented below. The fragment beginning from the start codon of *PA3459* is also included to show the localization of regulatory elements of *PA3458*p. (**B**) Relative expression of *PA3458* in PAO1161 in different phases of growth (OD_600_ ≈0.5, 1.0, 2.0, or >2.0 after 24 h). Relative expression presented in comparison to reference gene *nadB* and normalized to the level of PA3458 expression in the logarithmic phase (OD_600_ ≈0.5). Data represent mean expression from three biological replicates. (**C**) RT-qPCR analysis for chosen genes in Δ*PA3458* and WT tested in late logarithmic phase (OD_600_ ≈1.0). Mean fold change from three biological replicates is presented. The *nadB* was used as a reference gene. (**D**) Regulation of *PA3458* and *PA3459* promoter in PAO1161 WT or Δ*PA3458* in late logarithmic phase (OD_600_ ≈1.0). Data indicate mean β-galactosidase activity ±SD. * *p* < 0.01 in Student’s two-tailed t-test. (**E**) Regulation of *PA3459* and *PA3458* promoters by PA3458, assayed in *E. coli* Δ*lac*. Cells were transformed with pairs of vector pCM132 and derivatives and vector allowing expression of *PA3458* or the empty control vector. β-galactosidase activity was assayed in five independent transformants grown in medium with or without 0.2% arabinose.

To identify the DNA sequence preferentially bound by PA3458, a search for recurring DNA motifs was performed using 319 sequences corresponding to ±100 bp around summits of peaks with FE > 5 using MEME-ChIP [49] with the “zero or one occurrence per sequence” option (Table S4). The 15 bp sequence, with consensus TTHGNASDSRAARDA, hereafter called motif A, was obtained based on 306 sequences from 319 used in the analysis (Figure 4E, Table S3). The motif demonstrated conserved positions with preferred base pairs at positions 1, 2, 4, 11, 12, and 15. Conducting a similar analysis with nine PA3458 bound loci, upstream of PA3458 regulated genes (marked by a gray background in Table S4), yielded a similar motif (hereafter called motif A’) with the consensus sequence TTTCAGTTGGAAGCA (Figure 4F, Table S3). The motif was based on eight sequences out of nine used in the analysis. Motif A’ was not identified in *PA1270* promoter; however, this region encompassed a sequence matching the more general motif A (Figure 4G). Motifs identified in 306 peaks from the 319 analyzed are presented in Table S3. The putative binding motifs of PA3458 were located between predicted -10 boxes of promoter sequences and the start codon for *PA3459* (Figure 5A), *PA2664,* and *PA2015*; upstream of −35 sequence (*PA5170*, *PA5100*) or in a region encompassing −35 and/or −10 box of a predicted promoter sequence (*PA2264*, *PA1270*). PA3458 binding to these positions of promoter could potentially modulate the action of the RNA polymerase, hence influencing gene expression.

### 2.5. Regulation of Gene Expression by PA3458

To select conditions for testing the regulatory properties of PA3458 in *P. aeruginosa* cells, the RT-qPCR analysis of *PA3458* level was conducted using RNA from cells harvested at different growth stages. The highest expression of *PA3458* was observed in the late logarithmic phase (OD_600_ ≈ 1.0), indicating a possibility that in this phase, the action of PA3458 might be the most relevant and needed (Figure 5B). The conditions of late logarithmic phase were further exploited in the RT-qPCR analysis to quantify the transcripts level of chosen genes in Δ*PA3458* and WT *P. aeruginosa* cells. Analysis of *PA3459*, *PA3461*, *PA5170*, *PA2204*, and *PA4352* transcripts level in *PA3458-*deficient cells showed increased expression relatively to WT cells, while the two other tested genes *PA1270* and *PA2252* exhibited the decreased expression (Figure 5C). Importantly, for all tested genes, the opposite effect of change of their expression than those observed under conditions of PA3458 excess tested in RNA-seq was observed, confirming the role of PA3458 in their regulation (Table 1).

To verify further the regulatory action of PA3458, the *PA3459* promoter region was selected. *PA3459* is the first gene of the predicted operon *PA3459*–*PA3461*, which is located divergently to the *PA3458* gene in *P. aeruginosa* genome (Figure 1A) and showed the decrease in expression in response to PA3458 (Table 1). The divergent promoter *PA3458*p was also examined to assess the possible autoregulation of *PA3458*. The two promoter regions were cloned into the probing vector pCM132 carrying promoter-less *lacZ* (Table A1) [51]. The scheme of *PA3458* and *PA3459* promoter sequences is shown in Figure 5A. The potential binding motif of PA3458 is located 11 bp downstream of −10 sequence and 76 bp upstream of the start codon in *PA3459*p and 82 bp down-stream of −10 sequence and 51 bp upstream to the start codon of *PA3458*.

The pCM132 derivatives pKKB2.31 (*PA3458*p*-lacZ*) or pKKB2.32 (*PA3459*p*-lacZ*) were introduced into *P. aeruginosa* PAO1161 and Δ*PA3458* mutant, and the activity of promoters in the late logarithmic phase (OD_600_ ≈1.0) was tested (Figure 5D). The activity of *PA3459*p was hardly detected and at least 2-fold lower in comparison with *PA3458*p activity in PAO1161 (Figure 5D). However, the *PA3459*p activity was significantly higher in the Δ*PA3458* mutant in comparison with the WT strain, indicating possible promoter de-repression in the absence of PA3458. Interestingly, the activity of *PA3458*p was also higher in the Δ*PA3458* mutant in comparison with the WT strain, which suggests the autoregulatory function of PA3458.

Concomitantly, to check whether PA3458 regulates *PA3458*p and *PA3459*p, *E*. *coli* Δ*lac* cells were transformed with pCM132 derivatives, pKKB2.31 (*PA3458*p*-lacZ*) or pKKB2.32 (*PA3459*p*-lacZ*) together with pKKB2.11 (*araBADp-PA3458*), which are used for the production of PA3458 or the corresponding empty vector. The β-galactosidase activity measurements in extracts from stationary *E. coli* Δ*lac* cells showed that under tested conditions, the activity of *PA3458*p was at least two times higher than that of *PA3459*p (Figure 5E). When PA3458 was produced, a similar trend was observed. The overproduction of PA3458, by the addition of arabinose to the cultures, had no effect on the expression from *PA3458*p, but it significantly diminished the *PA3459p-lacZ* expression, indicating the repression of *PA3459*p by PA3458.

### 2.6. Phenotypic Characterization of PAO1161 ΔPA3458, ΔPA3459, and ΔPA3459–PA3461 Strains

Our studies point out the role of PA3458 in gene expression regulation in *P. aeruginosa* (Tables S1–S3), including negative regulation of the *PA3459* gene, which is part of the *PA3459*–*PA3461* operon that is transcribed divergently to *PA3458*. This operon encodes asparagine synthase (PA3459), GNAT-family acetyltransferase (PA3460), which is involved in the production of osmoprotectant N-acetylglutaminylglutamine amide (NAGGN) and hypothetical glutamyl aminopeptidase (PA3461) [9,52]. Previously, comparative microarray analysis showed that the *PA3459*–*PA3461* genes but not *PA3458* were up-regulated in *P. aeruginosa* cells in response to osmotic stress [9]. Concomitantly, analysis of the growth of *P. aeruginosa* Δ*PA3459* and Δ*PA3460* mutants in a medium with either 0.5 M NaCl or 0.7 M sucrose, representing conditions of osmotic stress showed that the absence of these genes negatively affects the growth of the cells in these conditions [9].

**Figure 6:**
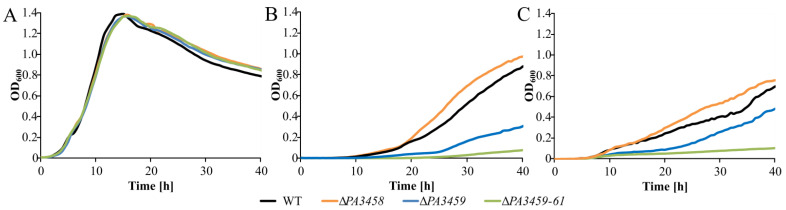
Phenotypic characterization of *P. aeruginosa* mutants in *PA3458*–*PA3461* genes. Growth of *P. aeruginosa* PAO1161 derivatives (WT, Δ*PA3458,* Δ*PA3459,* and Δ*PA3459*–*PA3461*) in minimal A medium with glucose (**A**) and supplemented with 0.5 M NaCl (**B**) or 0.7 M sucrose (**C**). Data represent mean OD_600_ from six cultures. Standard deviations were less than 15% and are not shown for clarity.

To investigate the significance of the PA3458 mediated regulation of *PA3459*–*PA3461* operon under osmotic stress, the Δ*PA3458*, Δ*PA3459,* and Δ*PA3459*–*PA3461* PAO1161 mutants, as well as WT, were cultivated in minimal A medium with or without 0.5 M NaCl or 0.7 M sucrose. No visible changes in bacterial growth between WT PAO1161 and mutants were observed in MA medium without NaCl or sucrose (Figure 6A). However, under conditions of osmotic stress, either in the presence of 0.5 M NaCl or 0.7 M sucrose, the Δ*PA3458* mutant showed repetitively better growth than WT, while the Δ*PA3459* and Δ*PA3459*–*PA3461* mutants grew much slower, which was likely caused by the impaired production of NAGGN (Figure 6B,C). The effect of faster growth observed for PAO1161 Δ*PA3458* strain under osmotic stress in comparison to WT cells might be explained by the higher expression of *PA3459*–*PA34561* in mutant cells, which is due to the lack of repression by PA3458, which allows better adaptation to osmotic stress. Interestingly, since one of the roles PA3458 plays in *P. aeruginosa* is the repression of the *PA3459*–*PA3461* operon, it seems that under standard, non-osmotic stress conditions, the repression of *PA3459*–*PA3461* operon is a more favourable state than its constant expression. In addition, the *PA3459* mutation has less effect on growth than the deletion of *PA3459*–*PA3461* operon, indicating that the inactivation of one gene is less deleterious that deletion of the whole operon for the cell at tested conditions.

### 2.7. Distribution and Evolutionary Conservation of PA3458–PA3461 Cluster in Bacteria

**Figure A3:**
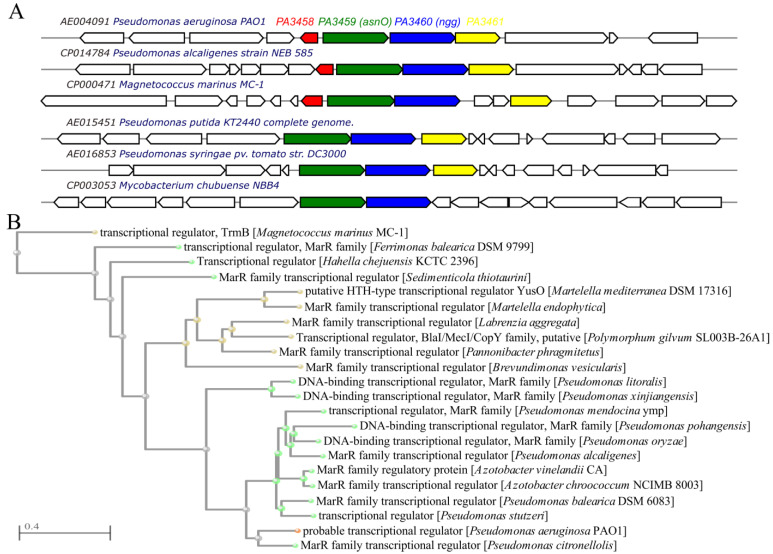
The occurrence of *PA3458*–*PA3461* gene cluster in bacteria. (**A**) MultiGeneBlast comparison [53] of clustered orthologs of *PA3458*–*PA3461* in representative and reference bacterial genomes included in the RefSeq database (release 91) [29]. Overall, 21 gene clusters were identified, and only selected results are presented (see also Table S5). (**B**) Phylogenetic tree of PA3458 orthologs constructed using CoBaltDB [90].

Previous studies showed that homologs of *PA3459* and *PA3460* genes, the *asnO-ngg* cluster, and their organization are conserved among many divergent bacterial species [52]. The presence of genes encoding orthologues of *PA3458*–*PA3461* together with a MarR-type transcriptional regulator similar to PA3458 was analyzed in available bacterial genomes using MultiGeneBlast [53]. This analysis yielded 22 genomes encoding orthologs of all four proteins with the same (except one *Magnetococcus marinus* MC-1) genes arrangement (Figure A3, Table S5). Fifty-two genomes encode the operon without regulatory genes, and 17 (mainly *Mycobacterium* sp.) code for *PA3459*–*PA3460* (Table S5). The 31–71%, 56–83%, 51–82%, and 49–84% identity of amino acid sequences was observed for homologues of PA3458, PA3459, PA3460, and PA3461, respectively, indicating the strong evolutionary conservation of enzymes and a bit lower for the regulator.

Nine strains encoding the *PA3458*–*PA3461* cluster belong to the genus *Pseudomonas* and seven are classified to the alpha-proteobacteria (Table S5). The identified strains represent different lifestyles, including pathogenic bacteria (plant or human) or strains isolated from an environment with high salinity such as seawater or saline soil. Interestingly, the *PA3458*–*PA3461* cluster was not conserved in so-called “honorary Pseudomonads”, which are species sharing similar metabolism and lifestyles although phylogenetically classified at some distance, e.g., in beta-proteobacteria from the *Burkholderia* or *Ralstonia* species, which often exchange genetic material with *Pseudomonas* bacteria [54]. The cluster was identified in some *Pseudomonads*, *Azotobacter* and alpha-proteobacteria from high-saline habitats, pointing out the need of osmoprotection and osmoadaptation functions in inhabited environments and the pressure to preserve genes encoding them.

When the genes encoding the orthologs of the PA3458 transcriptional regulator from 21 strains (Table S5) were aligned and their evolutionary distance was analyzed (Figure A3), the most similar gene to PA3458 was the TR from *P. citronellolis* SJTE-3, which is a strain that was isolated from sludge.

These results indicate that the presence of genes encoding PA3458–PA3461 proteins is not unique to PAO1/ PAO1161 or *Pseudomonas* sp. and occurs in other bacteria. For some of them, the correlation of the gene cluster occurrence with living in a high salinity environment could be noticed.

## 3. Discussion

The PA3458 protein belongs to the MarR family of transcriptional regulators [19]. In this study, the transcriptional profiling of cells overproducing PA3458 was performed, indicating 133 genes with altered expression. Concomitantly, more than 300 binding sites scattered in *P. aeruginosa* genome were identified, which highlights the great modulatory and/or regulatory potential of the PA3458 protein and may partially explain why its overproduction acts negatively on *P. aeruginosa* cells, leading to a cessation of bacterial growth.

Comparison of the genes with altered expression in response to PA3458 excess and PA3458 binding sites pointed out nine identified PA3458 binding sites upstream of regulated genes. The genes, potentially directly regulated by PA3458 encode proteins with a predicted function in stress response e.g., PA2664 (Fhp), PA2665 (FhpR), PA1429, PA1270, PA4352, PA2264, and amino acid metabolism PA3459, PA5170 (ArcD), PA5100 (HutU), PA2015 (LiuA), PA2016 (LiuR), and PA0866 (AroP2).

Among these, the PA3458 binding site with the highest fold enrichment was found in the promoter region of the *PA3459*–*PA3461*. RNA-seq analysis showed significantly decreased expression of *PA3459*–*PA3461* genes in response to PA3458 excess (Table 1), which indicates that PA3458, similar to many other TRs [55], regulates the divergent operon. The *PA3459*–*PA3461* operon encodes proteins involved in the production of a potent osmoprotectant: N-acetylglutaminylglutamine amide (NAGGN), conferring bacterial cells resistance to osmotic stress [9,52].

The PA3459 and PA3460 are homologues of AsnO and Ngg proteins from *S. meliloti* [52]. They are involved in a non-ribosomal synthesis of dipeptide NAGGN. In the first step, the Ngg catalyzes both the N-acetylation of one glutamine and the formation of a peptide bond with a second glutamine, producing the intermediate—NAGG—N-acetylglutaminylglutamine [52]. The AsnO transfers the amide nitrogen of another free glutamine to the second glutamine of NAGG, creating NAGGN [50]. This compound was detected in osmotically stressed cultures of *P. aeruginosa*, along with known osmoprotectants such as glutamate or trehalose [56]. At higher NaCl concentrations, NAGGN became the dominant osmolyte in *P. aeruginosa* [56].

MultiGeneBlast analysis showed that analogous genes arrangement for orthologs of a MarR-type regulator (PA3458) and divergent operon was found in at least 21 other strains with different lifestyles, including pathogens (Figure A3; Table S5). Interestingly, clustered genes encoding orthologs of *P*. *aeruginosa* PA3459–PA3461 were found in 52 other bacterial genomes. A closer inspection of their neighboring genes did not indicate the presence of transcriptional regulators; hence, it is likely that they are under the regulation of other factors encoded *in trans*. In *P. aeruginosa* cells, the highest mRNA level of PA3458 was detected in cells from the late logarithmic phase of growth. Our studies demonstrated the role of PA3458 in *PA3459*–*PA3461* repression. Why do these genes need to be repressed and tightly controlled in the cell? The simplest answer is that gene expression control is the most economical way to save resources. The enzymes encoded by *PA3459*–*PA3461* to produce NAGGN consume three glutamines and one acetyl-CoA [52]. Thus, uncontrolled production of NAGGN may deplete the cellular glutamine pool, and its availability is crucial for many cellular processes.

PA3458 acts as a repressor of the *asnO-ngg* genes, allowing modulation of their expression depending on growth conditions and possibly regulating the NAGGN pool during adaptation to osmotic fluctuations. Such variations could be also the part of intracellular changes accompanying the transition between the logarithmic and stationary phases of growth. This may partially explain the changes in *PA3458* expression dependent on a growth phase (Figure 5B). A comparison of logarithmically and stationary growing WT PAO1161 cells indicated the lower expression of *PA3458* and concomitant higher expression of *PA3459*–*PA3461* in stationary phase cultures (Bartosik AA et al., in preparation). Interestingly, a similar expression pattern was observed for the *slyA* gene of *S. typhimurium*, which encodes the global regulator playing a crucial role in survival in the intra-phagosomal environment and in resistance to macrophage killing [43,57].

In the proposed model of action, the PA3458 protein binds to the promoter region of *PA3459* gene and represses its expression. This negative regulation is released when the conditions of growth change for example by increasing the osmolarity of an environment, allowing the production of NAGGN osmoprotectant. It helps sensitively adjust the expression of the *asnO* and *ngg* in the cells. We hypothesize that the increasing concentration of ions, e.g., Na^+^, K^+^, and/or binding of unknown ligand causing allosteric inhibition may modulate PA3458 activity, e.g., its inability to interact with DNA, which triggers de-repression of the *PA3459*–*PA3461* operon. Many MarR-type transcriptional regulators are allosterically inhibited [55]. Additionally, the changes in DNA structure and/or topology, e.g., supercoiling, which are observed under osmotic stress conditions or the transition to stationary phase may also influence PA3458 interaction with DNA [58,59,60]. The specific requirements for DNA binding by PA3458 are supported by the observation that despite numerous tries, setups, and multiple conditions tested, we failed to demonstrate any PA3458 binding to DNA using electrophoretic mobility shift assays (EMSA, data not shown).

The action of PA3458 is not limited to one target. More than 300 PA3458 binding sites were identified in *P. aeruginosa* genome, indicating broad protein interactions with DNA. The motif sequence bound preferentially by PA3458 was identified, and it is characterized by AT-rich regions at both ends resembling in part a palindromic structure with a variable center (Figure 4E,F). A more specific motif sequence recognized by PA3458 was identified, when PA3458 binding sites with the highest fold enrichment in ChIP-seq analysis and detected in promoter regions of regulated genes were analyzed. This may suggest the evolutionary pressure to maintain preferred nucleotide positions in sequences recognized by PA3458 to exert a stronger effect on gene regulation. Although the motif is not strictly palindromic it resembles in part a palindromic structure with preferred double T and A base pairs at positions 1,2 and 11,12, respectively of the proposed motif with a more flexible center in between these positions and additional extension with conserved A at position 15. In motif A’, more positions tend to be conserved, and the partial palindromic structure within the first 12 base pairs positions of the motif is even more evident, as presented by underlined positions marked by // indicating the plane of symmetry in the consensus sequence TTTCAG//TTGGAA GCA presented in Figure 4F. The identified motif resembles the MarR-type regulator SlyA binding site TTAGCAAGCTAA [43]. It is worth mentioning that also non-perfectly palindromic sequence motifs bound by MarR-type transcriptional regulators were found e.g., for MalR from *Corynebacterium glutamicum*, consensus TTnAAnnnTCAA [61]; HpaR from *Xanthomonas campestris*—consensus [G/T]CAACAATT[C/T]TTG [62] or CosR from *Vibrio parahaemolyticus*—consensus TTTGA-NN-TCTAA [63].

For some PA3458 binding sites identified in the promoters of down-regulated genes (*PA3459*, *PA2664*, *PA2015*, *PA4352*, *PA1429*), the motif is located upstream of the start codon and often downstream of -10 promoter sequence; thus, it is in a position to interfere with RNA polymerase action. In the case of *PA2264* and *PA1270* promoters potentially stimulated by PA3458, the binding motif encompass -35 or -10 promoter sequences, respectively, possibly positively influencing RNAP activity. It is not excluded that regulator binding to the site in the coding region may also exert the effect on gene expression regulation as exemplified by *PA1596*, *PA2247*, or *PA0866* as target genes of PA3458, but the molecular mechanism of gene expression control in this way requires further studies.

The weaker PA3458 interactions with less conserved binding sites scattered in *P. aeruginosa* genome were also detected, and it is not excluded that under special growth conditions, such interactions may also be part of the regulatory network [55]. Many MarR regulators are biosensors and bind to DNA or dissociate from it under specific conditions e.g., changes in oxidation level, pH, or sensing chemical signals [19]. These data indicate that specific DNA binding by PA3458, similar to other MarR-type transcriptional regulators, may require not only specific sequence but also other factors.

The study showed that PA3458 may potentially control the expression of genes other than the divergently encoded operon. The *fhp* (*PA2664*) gene with a strong PA3458 binding site in the promoter region (FE > 26) was 42-fold down-regulated in PA3458+ cells. It encodes the flavohemoprotein Fhp necessary to protect bacterial cells from nitrosative stress by detoxifying NO to nitrate [64,65] and induced in response to NO [66]. The expression of *fhpR* (*PA2665*) encoded divergently to *fhp* and encoding the transcriptional activator of *fhp* [64] was also 2-fold down-regulated in PA3458+.

The other gene whose expression was significantly diminished in response to PA3458 is *PA1429* encoding a probable cation-transporting P-type ATPase. The PA1429 inhibits *Pseudomonas* quinolone signal (PQS) synthesis and influences bacterial motility, biofilm formation, or virulence [67].

The PA3458 binding site was detected in the promoter region of the *arcDABC* operon, which also responded by a decrease in expression in response to PA3458. The *arc* operon encodes proteins involved in anaerobic arginine catabolism in *P. aeruginosa* [68,69]. The deletion mutant in the *arcD* gene exhibited increased bacterial motility, biofilm formation, and virulence in a mouse model of acute lung infection [69]. Arginine is shown as a killing enhancer by ciprofloxacin and tobramycin under anaerobic, but not aerobic, growth conditions [68,69]. This indicates that regulation of the stress response by PA3458 may extend beyond the control of NAGGN production.

In *P. aeruginosa*, the extensive transcription regulatory network allows complex and precise response to changes in the environment. It is not surprising that some PA3458-dependent genes are also parts of other regulons and are co-regulated by other regulatory proteins: e.g., the *arcDABC* operon by a nitrate-responsive NarX–NarL regulator [70]; the *pauA4* by PauR [71]; the *fhp* by PA3697 [72]; the *liuR, liuA* by Hfq–Crc [73] or PA4352 by Anr [74]. The analysis presented here adds a new player to the existing network.

To summarize, this study showed that the representative of the MarR-type regulators PA3458 is involved in gene expression control in *P. aeruginosa.* Many binding sites of the protein were identified in *P. aeruginosa* genome predisposing PA3458 to play a role as the global regulator, with one of the direct targets engaged in the production of bacterial osmoprotectant NAGGN. The activity of PA3458 and its target genes may play a role in an adaptation of bacterial cells to changing growth conditions, including osmotic stress.

## 4. Materials and Methods

### 4.1. Growth Conditions, Bacterial Strains, and Plasmids Manipulations

Bacterial strains and plasmids used in this study are listed in Table A1. *P. aeruginosa* PAO1161 strain [48], a derivative of PAO1 possessing mutations in *leuA*, *PA2735,* and *rpoB* genes connected with *leu*^−^, *r*^−^, Rif^R^ phenotypes, respectively, was used in most experiments except for those conducted in minimal media, in which *leu*^+^ derivative of PAO1161 was used (Table A1). The PAO1161 strain carries ICE*Pae*1161, which is a functional PAPI-1 family integrative conjugative element conferring mercury resistance [48], but this element should not have influence on the outcome of experiments described in this manuscript.

Bacterial strains were grown in Luria–Bertani (LB) broth, minimal media MA, and M9 or on LB plates containing 1.5% (*w*/*v*) agar [75]. For the selection of plasmids, LB medium was supplemented with appropriate antibiotics: kanamycin (50 μg mL^−1^ for *E. coli*, 500 μg mL^−1^ in solid media, and 250 μg mL^−1^ in liquid media for *P. aeruginosa*); benzylpenicillin sodium salt (300 μg mL^−1^ in solid media and 150 μg mL^−1^ in liquid media for *E. coli*); carbenicillin (300 μg mL^−1^ for *P. aeruginosa*); rifampicin (300 μg mL^−1^ for *P. aeruginosa*); chloramphenicol (10 μg mL^−1^ for *E. coli*, 150 μg mL^−1^ for *P. aeruginosa*). Plasmid DNA isolation, manipulation, and transformation into competent cells of *E. coli* was conducted prepared by the CaCl_2_ method [76].

Cultures for RNA-seq, ChIP-seq, and RT-qPCR analyses were conducted in flasks closed with a cotton plug, filled with medium to 20% of volume. Cultures were incubated at 37 °C with shaking 200 rpm.

Kinetics of growth were analyzed at 37 °C upon 100-fold dilution of the overnight cultures in LB, washed with the appropriate medium in LB, minimal medium MA, or M9 with various additives, such as 0.25% citrate or 17 mM glucose as a carbon source and 0.5 M NaCl or 0.7 M sucrose [9,76]. Bacterial growth in 96-well plates was monitored by measurements of optical density at 600 nm (OD_600_) using a Varioskan Lux Multimode Microplate Reader and SkanIt RE 6.0.2 software (Thermo Fisher Scientific, Waltham, MA, USA).

The *P. aeruginosa PA3458*, *PA3459*, or *PA3459*–*PA3461* deletion mutants (Δ*PA3458*, Δ*PA3459*, or Δ*PA3459*–*61*) were constructed by allele exchange using the pAKE600 suicide vector derivatives [77]: pKKB2.61, pKKB2.62, and pKKB2.63 (Table A1). The upstream and downstream DNA fragments of mutated regions were amplified using selected primers (1#/2# pair and 3#/4# pair for *PA3458*, 5#/6# pair and 7#/8# for *PA3459* or 5#/6# pair and 9#/10# for *PA3459*–*PA3461*) (Table A2). The PCR products, corresponding to upstream and downstream regions were digested with BamHI, HindIII and HindIII, EcoRI, respectively, and both fragments were ligated with EcoRI, BamHI digested pAKE600. *E. coli* S17 strain, carrying suicide plasmids was conjugated with the recipient strain *P. aeruginosa* PAO1161 (Rif^R^). Putative cointegrants were selected on LB agar with rifampicin and carbenicillin. The vector removal by second recombination was conducted by growing the cells in LB with 10% sucrose. Colonies with exchanged alleles were selected by PCR.

Motility (swimming, swarming) assays were performed as described previously [38,76] for PAO1161 and Δ*PA3458* mutant for 24 h at 37 °C. Plates were standardized by using the same volume of each medium.

Biofilm analysis was performed on LB or minimal medium with citrate. Cultures were grown for 24 h and 48 h at 37 °C, respectively. The measurements were carried out according to the previously described method [76].

### 4.2. Construction of Expression Vectors and Protein Purification

To obtain vectors allowing the production of PA3458 His_6_-tagged at the N- or C-terminus, the gene was cloned in pET28a (Novagen). To construct the *his_6_*-*PA3458* fusion, PA3458 was amplified by PCR with the use of 11#/12# primer pair and PAO1161 genomic DNA as a template. The product was digested with EcoRI, SacI, and ligated with EcoRI, SacI digested pET28a to obtain pKKB2.21. Similarly, to obtain *PA3458*-*his*_6_ fusion, PCR fragment amplified using a 13#/14# primers pair was digested with NcoI, HindIII, and ligated with pET28a digested with NcoI, HindIII to yield pKKB2.22.

Overproduction of His_6_-PA3458 and PA3458-His_6_ prior purification was carried out in *E. coli* BL21 carrying pKKB2.21 or pKKB2.22, respectively. The overnight cultures of transformants were diluted 1:50 in 500 mL LB with kanamycin and grown for 1 h at 37 °C. Protein expression was induced with 0.5 mM isopropyl β-D-1-thiogalactopyranoside (IPTG), and the cultures were grown at 37 °C for 4 h. Cells were harvested by centrifugation, and the pellet was resuspended in LEW buffer (50 mM NaH_2_PO_4_, 300 mM NaCl pH = 8) with 1 mM protease inhibitor phenylmethylsulfonyl fluoride (PMSF) and 1 mg ml^−1^ lysozyme. After 0.5 h incubation on ice, the mixture was sonicated and cleared by centrifugation. The supernatant was collected and applied on Ni-agarose columns (Ni-TED 1000 Protino, Marchel&Nagel), followed by washing using 20 mL LEW and eluated using 4 × 1 mL LEW with 250 mM imidazole. The purification procedure was monitored by sodium dodecyl sulfate (SDS) polyacrylamide gel electrophoresis (PAGE) with a Pharmacia PHAST gel system. Elution fractions with the highest concentration of proteins were dialyzed against LEW buffer with 10% glycerol. Small aliquots of the purified protein were stored at −80 °C for further analysis.

To obtain an expression vector allowing expression in *P. aeruginosa*, the *PA3458* gene was amplified using 11#/12# primer pair, EcoRI, SacI digested, and ligated with EcoRI, SacI digested pKGB8, downstream of arabinose regulated *araBAD*p to yield pKKB2.11. This plasmid was introduced to *P. aeruginosa* PAO1161 and *E. coli* DH5α to test the effects of protein overproduction at various concentrations of the inductor (arabinose).

To obtain *PA3458-flag* translational fusion, the *PA3458* sequence was cloned using the PCR amplified fragment with #11/#15 primers and ligated to pKAB20B vector (with *flag*-tag) after EcoRI, BamHI digestion. Then, the *PA3458-flag* fragment was excised using EcoRI and SalI and then cloned into pKGB8 to obtain pKKB2.12.

### 4.3. Glutaraldehyde Crosslinking

The oligomerization state of purified His_6_-*PA3458* and *PA3458*-His_6_ was assayed by crosslinking using glutaraldehyde in concentration up to 0.05% in a buffer composed of 50 mM *N*,*N*-Bis(2-hydroxyethyl)glycine-NaOH (BICINE-NaOH); 0.1 mM ditiotreitol (DTT), and 0.4 M NaCl. For each 20 µL reaction, 2 µg of protein was used. After 20 min, the reaction was stopped by adding ethanolamine-HCl (pH 8) to a final concentration of 0.14 M. Samples were analyzed using SDS-PAGE, and the protein was visualized by immunodetection, using anti-His antibodies after the transfer onto a nitrocellulose membrane.

### 4.4. SEC-MALS Analysis

Size exclusion chromatography coupled to multi-angle light scattering (SEC-MALS) analysis was performed using a high-performance liquid chromatography (HPLC) instrument (1260 Infinity LC, Agilent Technologies Inc., Santa Clara, CA, USA) equipped with a UV detector, a MALS detector (DAWN HELEOS II, Wyatt Technology, Santa Barbara, CA, USA), and a differential refractometer (Optilab T-rEX, Wyatt Technology, Santa Barbara, CA, USA). Then, 100 µL of 1 mg ml^−1^ samples were loaded onto a Superdex 200 Increase 10/300 column (GE Healthcare, Milwaukee, WI, USA) equilibrated with LEW buffer. Absorption at 280, 254, and 215 nm was monitored during SEC. Samples were run at room temperature at a flow rate of 0.5 mL min^−1^. The results were analyzed using ASTRA v. 6 software (Wyatt Technology, Santa Barbara, CA, USA) in accordance with the manufacturer’s instructions.

### 4.5. RNA Isolation, RNA-seq, and RT-qPCR Analysis

Strains were obtained by transformation of PAO1161 with pKKB2.11 (*araBADp-PA3458*) or pKGB8 (*araBAD*p) plasmids (Table A1). Transformants were selected on LB plates supplemented with 150 μg mL^−1^ chloramphenicol and were verified by isolation of plasmid DNA and its digestion. After overnight growth, each of three cultures were diluted 1:100 into fresh LB supplemented with 75 μg mL^−1^ chloramphenicol and 0.02% arabinose. Cells were collected from 2 mL of cultures in the logarithmic phase of growth (optical density at 600 nm of 0.4–0.6) and mixed with 4 mL of RNAprotect Bacteria Reagent (Qiagen, Hilden, Germany). RNA was isolated using the Qiagen RNeasy Mini Kit, according to the manufacturer’s instructions. Isolated RNA was treated with a DNA-free DNA Removal Kit (Invitrogen, Thermo Fisher Scientific, Waltham, MA, USA), and a lack of DNA contamination was checked by PCR. RNA concentration was determined using a μDrop plate of Varioskan Lux Multimode Microplate Reader and quality was checked using Bioanalyzer. Library preparation and sequencing were performed in the Genomed S.A., Warsaw, Poland. rRNA was depleted using Ribo-Zero™ rRNA Removal Kit (Bacteria) (MRZMB126, Illumina, San Diego, CA, USA) according to manufacturer instructions. Libraries were prepared according to instructions accompanying the NEBNext^®^ Ultra™ DNA Library Prep Kit for Illumina (E7370S, New England Biolabs, Ipswich, MA, USA).

Libraries were sequenced using standard Illumina protocols (NextSeq500, paired-end with 150 cycles for each read). Reads were quality-checked and filtered using FASTP version 0.20.0 [78] and mapped to *P. aeruginosa* PAO1161 genome (CP032126.1) using Bowtie2 version 2.3.4.3 [79] with default settings. The number of reads mapping to individual genes was counted using FeatureCounts v 2.0.1 (part of the Subread) with the -s2 option [80]. Differential expression analysis was conducted using edgeR ver 3.28.0 [81]. Raw data are available in the NCBI‘s Gene Expression Omnibus (GEO) database (http://www.ncbi.nlm.nih.gov/geo/, accesses on 20 February 2021) under accession number GSE167147.

For qRT-PCR analyses, cells from PAO1161 WT and Δ*PA3458* mutant cultures were collected from 2 mL of cultures at an optical density at 600 nm 0.5, 1.0, or 1 mL for cultures with an optical density at 600 nm of 2.0 or after 24 h. RNA for qRT-PCR was isolated identically as for RNA-seq analysis. Reverse transcription was performed with 4 µg of RNA using the TranScriba Kit (A&A Biotechnology, Gdansk, Poland). qPCR was performed on a LightCycler 480 II System (Roche Molecular Diagnostics, Mannheim, Germany) using 5× HOT FIREPol EvaGreen qPCR Mix Plus (Solis Biodyne, Tartu, Estonia). Each 18 µL reaction contained 3.6 µL 5× reaction mix, 1 µL of five times diluted cDNA, and 1.5 µL of mixed 5 µM primers. The relative expression was determined by a comparison of crossing points (Cp) between the target and the reference gene (*proC* or *nadB*). Three technical repetitions were used for each primer pair. The ratio/fold change was calculated using Pfaffl’s formula [82].

### 4.6. Chromatin Immunoprecipitation with Sequencing (ChIP-seq)

ChIP-seq analysis was performed on PAO1161 Δ*PA3458* strain overproducing PA3458-FLAG (pKKB2.12) [PA3458-F+] as well as the strain carrying the empty vector pABB28.3 (background control) in LB containing 50 μg mL^−^^1^ chloramphenicol. Four independent overnight cultures from each strain were inoculated and diluted 1:100 in LB supplemented with 75 μg mL^−1^ chloramphenicol and 0.02% arabinose. Bacteria were grown at 37 °C until reaching the exponential phase with optical density at 600 nm (OD_600_) about 0.5.

ChIP protocol was based on a modified S. Schulz and S. Haussler protocol using Dynabeads Protein A [83]. The procedure was performed as described earlier until the step of sonication [84]. Lysate after sonication was thawed on ice, and 150 μL of each strain variant was incubated with 20 μL of magnetic beads coupled with protein A (Dynabeads Protein A, Invitrogen, 10001D), which was separated from original suspension using a Magnetic Separation Stand. The pre-clearing step was performed for 1 h at 4 °C with a rotation of the mixtures. Then, 50 μL of magnetic beads, separated from the suspension as above, was mixed with 6 μL of anti-FLAG mouse polyclonal antibodies (DYKDDDDK Tag polyclonal antibodies; PA1-985B; Invitrogen (Thermo Fisher Scientific, Waltham, MA, USA); 1 mg mL^−1^) diluted in 200 μL of PBS (phosphate buffered saline) with 0.05% Tween-20. Mixtures of magnetic beads and antibodies were incubated for 10 min at 4 °C with gentle rotation. Then, beads with bound antibodies were separated from the supernatant, washed once with 200 μL of the PBS with 0.05% Tween-20 solution, and stored on ice. Pre-cleared lysate was separated from the beads and added to the beads coated with antibodies. A mixture containing lysate and magnetic beads with antibodies was incubated at 4 °C for 20 min with mixing on a rotator. Then, beads were collected and washed as described earlier [84]. Elution was performed twice for 15 min in 50 μL at 65 °C in a thermoblock with shaking (1400 rpm). Elutions from 6 parallel reactions were pooled and then 30 µL pipetted for Western blot analysis. The rest of the obtained eluates were incubated with 8 μL of RNase A (100 mg mL^−1^, 19101, Qiagen, Hilden, Germany) for 30 min at 65 °C. Then, 40 μL of Proteinase K (20 mg mL^−1^, 19133, Qiagen) was added, and the samples were incubated for 1 h at 50 °C followed by overnight incubation at 65 °C. Next, 40 μL of Proteinase K was added, and the samples were again incubated for 1 h at 50 °C. Subsequently, 24 μL of 3 M sodium acetate (pH 5) was added, and the volume was adjusted to 700 μL using water. DNA purification was performed using a Qiaquick Qiagen PCR purification Kit (Qiagen, Hilden, Germany) according to the manufacturer’s instructions. The DNA was stored at −20 °C. Purified DNA from ChIP performed with empty vector strain was included as a background control.

Sequencing of ChIP samples was performed in the Laboratory of DNA Sequencing and Oligonucleotides Synthesis of Institute of Biochemistry and Biophysics Polish Academy of Sciences in Warsaw, Poland. NGS libraries were constructed using a QiaSeq Ultralow Input Library kit (Qiagen, Hilden, Germany). Samples were quality checked on 1% agarose gel, and concentration was measured using a qPCR KAPA Library Quantification Kit (Roche Holding AG, Basel, Switzerland). Libraries were sequenced using standard Illumina protocols.

Reads were quality-checked and filtered using FASTP version 0.20.0 [78]. Reads were mapped to the *P. aeruginosa* PAO11161 genome (CP032126.1) using Bowtie2 version 2.3.4.3 [79] using default settings. Obtained *.sam files were sorted (samtools sort -n), run through samtools fixmate with the -m option, again sorted (samtools sort), and duplicates were marked with samtools markdup. Samtools ver. 1.9 was used [85]. The files were indexed and used to generate coverage *.bigwig files, which were normalized to 1× sequencing depth (RPGC), without binning and smoothing, using the bamCoverage tool ver 3.3.0, included in deepTools [86]. ChIP-seq peaks were called for merged ChIP replicates using MACS2 ver 2.1.2 [87] with default options for paired-end BAM files and 0.05 as the false discovery rate (FDR) adjusted p-value cut off. Peaks annotation and visualization of the coverage data were performed using custom R scripts. Raw data are available in the NCBI‘s Gene Expression Omnibus (GEO) database (http://www.ncbi.nlm.nih.gov/geo/; accessed on 20 February 2021) under accession number GSE167146.

### 4.7. Construction of Promotor-lacZ Transcriptional Fusions and Promoter Activity Testing

To test promoter activity in *E. coli* and *P. aeruginosa*, an RK2 derivative, the pCM132 plasmid with a promoterless *lacZ* reporter gene was used [51]. To construct pCM132 derivatives carrying *PA3458p-lacZ* and *PA3459p-lacZ*, DNA fragments were amplified by PCR using #17/18# or 19#/20# primer pairs, respectively, and digested with EcoRI, BamHI to ligate with EcoRI, BglII cut vector to obtain pKKB2.31 (*PA3458p-lacZ*) and pKKB2.32 (*PA3459p-lacZ*).

*E. coli* DH5α Δ*lac* was transformed with pairs of plasmids: pCM132m/pKGB8 (empty vectors as a control); pCM132m/pKKB2.11 (*araBADp*-PA3458); pKKB2.31 (*PA3458p-lacZ*)/pKKB2.11; pKKB2.31/pKGB8; pKKB2.32 (*PA3459p-lacZ*)/pKKB2.11; pKKB2.32/pKGB8. The β-galactosidase activity was tested in extracts from stationary cells conducted without and with 0.02% arabinose at 37 °C in LB containing kanamycin and chloramphenicol.

The β-galactosidase activity was also tested in extracts from PAO1161 WT and Δ*PA3458* mutant carrying the empty vector (pCM132m), *PA3458p-lacZ* (pKKB2.31) or *PA3459p-lacZ* (pKKB2.32) grown at 37 °C in LB containing kanamycin.

### 4.8. Bioinformatic Analysis

A comparison of the HTH domain of chosen MarR-type regulators was performed using Clustal Omega [39]. Initial model of homodimer was generated by SWISS-MODEL [40]. DNA docking was performed in HDOCK SERVER [41]. Snapshots of a model were taken in UCSF Chimera software [88]. The DNA binding motifs were identified using MEME-ChIP version 5.3.0 [50] using 200 bp around 319 or 9 PA3458 peak summits (Table S4). Clustered orthologs of *PA3458*–*PA3461* were identified in 1748 reference genomes from Refseq database (Release 91) using MultiGeneBlast [53]. The -35/-10 boxes of promoter sequences were predicted using BPROM [89].

## 5. Conclusions

The PA3458 is the representative of the MarR-type regulators, which are involved in gene expression control in *P. aeruginosa.* Many binding sites of the protein were identified in *P. aeruginosa* genome, predisposing PA3458 to play a role as the global regulator, with one of the direct targets engaged in the production of bacterial osmoprotectant NAGGN. The transcriptional profiling showed relatively high expression of *PA3458* in *P. aeruginosa* cells, except the late stationary phase, with the highest mRNA level detected in cells from the late logarithmic phase of growth. The activity of PA3458 and its target genes may play a role in the adaptation of bacterial cells to changing growth conditions, including osmotic stress. This is especially important in the light of infections caused by *P. aeruginosa* and changeable conditions prevailing during infection.

## Figures and Tables

**Table 1:** Selected PA3458 binding sites in PAO1161 genome in the proximity of genes showing altered expression in response to PA3458 overproduction.

Peak nr	Peak Summit	Fold Enrichment	Fold Change	Feature *	PAO1161 [D3C65_] ID	PAO1 ID	Gene Name	Gene Product
296	1650070	27.17	−3.62	promoter −	07825	PA3459		N-acetylglutaminylglutamine amidotransferase
			49.67	promoter +	07830	PA3458		MarR family transcriptional regulator
460	2502004	26.49	−42.63	promoter +	12095	PA2664	*fhp*	NO-inducible flavohemoprotein
			−2.05	promoter -	12090	PA2665	*fhpR* *(norR)*	nitric oxide reductase transcriptional regulator FhpR (NorR)
1148	5940238	23.15	−9.38	promoter +	28110	PA5170	*arcD*	arginine:ornithine antiporter
552	3022546	11.73	2.27	promoter −	14200	PA2264		gluconate 2-dehydrogenase subunit 3 family protein
758	4136054	18.41	5.03	promoter −	19465	PA1270		FUSC family protein
905	4819815	17.66	2.08	gene body	22785	PA4295	*fppA*	type 4b pilus Flp prepilin peptidase
913	4880773	16.55	−11.11	promoter −	23080	PA4352		universal stress protein
731	3960512	13.75	−15.23	promoter −	18645	PA1429		cation-transporting P-type ATPase
686	3777799	12.53	−3.87	gene body	17765	PA1596	*htpG*	molecular chaperone HtpG
598	3309346	11.61	−3.39	terminator +	15560	PA2016	*liuR*	MerR family DNA-binding transcriptional regulator
			−3.09	promoter +	15565	PA2015	*liuA*	isovaleryl-CoA dehydrogenase
770	4215905	8.68	−2.01	terminator −	19850	PA1197		hypothetical protein
1130	5864366	7.23	−6.09	promoter −	27730	PA5100	*hutU*	urocanate hydratase
195	1065438	6.33	−2.47	terminator +	05130	PA3971		PaaI family thioesterase
555	3042187	6.20	−3.60	gene body	14290	PA2247	*bkdA1*	3-methyl-2-oxobutanoate dehydrogenase (2-methylpropanoyl-transferring subunit alpha
860	4567471	6.11	−2.76	gene body	21640	PA0866	*aroP2*	amino acid permease

* “+”; “−“—coding strand.

## Data Availability

Raw data of RNA-seq and ChIP-seq are available in the NCBI‘s Gene Expression Omnibus (GEO) database (http://www.ncbi.nlm.nih.gov/geo/, accessed on 20 February 2021) under accession numbers GSE167147 and GSE167146, respectively.

## Supplementary Materials

The following are available online at https://www.mdpi.com/article/10.3390/ijms22083982/s1: Table S1: Results of RNA-seq analysis of PA3458+ vs. EV+ *P. aeruginosa* PAO1161 cells. List of genes with altered expression identified by comparison of transcriptomes of cells overproducing PA3458 with transcriptomes of cells carrying the empty vector (fold change (FC) ≤ −2 or ≥ 2, FDR adjusted *p*-value ≤ 0.05). Genes annotated only in PAO1161 strain but not in PAO1 are described as “not annotated”. Table S2: Results of ChIP-seq analysis. The table represents data for PA3458-FLAG ChIP-seq peaks identified in each ChIP repeat without peaks found in negative control, as identified by MACS2 analysis. FDR-adjusted p-value for all peaks was lower than 0.05. Table S3: Results of ChIP-seq analysis with cut-off FE > 5 (319 peaks). The table represents data for PA3458-FLAG ChIP-seq peaks identified in each ChIP repeat without peaks found in negative control, as identified by MACS2 analysis. FDR-adjusted p-value for all peaks was lower than 0.05. Motifs identified in summits of 306 ChIP-seq peaks, used to construct the logo of PA3458 binding site (motif A and A’-bolded). The PA3458 binding sequence motifs were identified using MEME-ChIP version 5.3.0 [49,50]. Table S4: Sequences of ChIP-seq peak summits with FE > 5. In gray are marked sequences used to search PA3458 binding motif A’. Table S5. Distribution and evolutionary conservation of PA3458–PA3461 cluster in bacteria identified using MultiGeneBlast [53].

## Appendix A

**Table A1:** Strains and vectors used in this study.

*E. coli* Strains		
DH5α	F^−^ (*φ80*d*lacZ*ΔM15) *recA1 endA1 gyrA96 thi-1 hsdR17* (r_k_^−^m_k_^+^) *supE44 relA1 deoR* Δ(*lacZYA–argF*)*U196*	[91]
BL21	F^−^ *ompT hsdS*_B_ (r_b_^−^m_b_^−^)*gal dcm* (*λ* DE3)	[91]
S17-1	*pro hsdR hsdM recA* Tp^R^ Sm^R^ ΩRP4-Tc∷Mu-Km∷Tn*7*	[91]
DH5α Δ*lac*	F- (80dlacZM15), *rec*A1, *en-d*A1, *gyr*A96, *thi*-1, *hsd*R17 (rk-mk+), *sup*E44, *rel*A1, *deo*R Δ(lacZYA-argF), U196	lab collection
***Pseudomonas aeruginosa*** **Strains**		
PAO1161 *leu*^−^	*leu*^−^ r^−^m^+^ Rif^R^	[38]
PAO1161 *leu*^−^ Δ*PA3458*	deletion of 462 bp fragment encompassing *PA3458* gene	this work
PAO1161 *leu*^−^ Δ*PA3459*	deletion of 1758 bp fragment encompassing *PA3459* gene	this work
PAO1161 *leu*^−^ Δ*PA3459*–*PA3461*	deletion of 4749 bp fragment encompassing *PA3459*–*PA3461* putative operon	this work
PAO1161 *leu*^+^	r^−^m^+^ Rif^R^	[48]
PAO1161 *leu*^+^ Δ*PA3458*	deletion of 462 bp fragment encompassing *PA3458* gene	this work
PAO1161 *leu*^+^ Δ*PA3459*	deletion of 1758 bp fragment encompassing *PA3459* gene	this work
PAO1161 *leu*^+^ Δ*PA3459*–*PA3461*	deletion of 4749 bp fragment encompassing *PA3459*–*PA3461* putative operon	this work
**Plasmids**		
**pAKE600**	Ap^r^; *ori*_MB1_*oriT*_RK2_, *sacB*	[77]
pKKB2.61	Ap^r^; pAKE600 derivative with 437 bp fragment encompassing up- and down-sequence of *PA3458*, amplified using 1#/2# and 3#/4# primers and cloned using BamHI, HindIII/HindIII, EcoRI	this work
pKKB2.62	pAKE600 derivative with 530 bp fragment encompassing up- and down-sequence of *PA3459*, amplified using 5#/6# and 7#/8# primers and cloned using BamHI, HindIII/HindIII, EcoRI	this work
pKKB2.63	pAKE600 derivative with 541 bp fragment encompassing up- and down-sequence of *PA3459*–*PA3461*, amplified using 5#/6# and 9#/10# primers and cloned using BamHI, HindIII/HindIII, EcoRI	this work
**pET28a**(**+**)	Km^r^; *ori* pBR322; *ori* f1; expression vector	Novagen
pKKB2.21	pET28a (+) derivative with 513 bp fragment, encoding His_6_-PA3458, amplified using 11#/12# primers and cloned using EcoRI, SacI	this work
pKKB2.22	pET28a (+) derivative with 483 bp fragment, encoding PA3458-His_6_, amplified using 13#/14# primers and cloned using NcoI, HindIII	this work
**pKGB8**	Cm^r^; IncA/C broad-host-range cloning vector, mob, *araC-pBAD*p	[46]
pABB28.3	pKGB8 derivative with *flag*	this work
pKKB2.11	pKGB8 derivative with 513 bp fragment, encoding PA3458, amplified using 11#/12# primers and cloned using EcoRI, SacI	this work
**pKAB20B**	Ap^r^; pUC19 derivative with *His_6_-mcs* (MunI, HindIII, NotI, XhoI, BamHI)-*flag*	this work
pKKB2.91	pKAB20B derivative with 483 bp fragment, encoding PA3458–FLAG, amplified using 11#/15# primers and cloned using NcoI, BamHI	this work
pKKB2.12	pKGB8 derivative with 618 bp fragment encoding PA3458-FLAG cloned using EcoRI, SalI cut from pKKB2.91	this work
**pCM132**	Km^r^; *oriV*_RK2_; *oriT*_RK2_; promoter less *lacZ* reporter gene	[51]
pKKB2.31	pCM132 derivative with 281 bp fragment amplified with primers 17#/18#, cloned using EcoRI, BamHI/BglII encompassing *PA3458* promoter (*PA3458*p)	this work
pKKB2.32	pCM132 derivative with 266 bp fragment amplified with primers 19#/20#, cloned using EcoRI, BamHI/BglII encompassing *PA3459* promoter (*PA3459*p)	this work

**Table A2:** Primers used in this study.

Nr	Starter Name	Use	Seqence
#1	3458mLF	*PA3458* gene deletion	gcggatccACGAAACTCTCCTGATATG (BamHI)
#2	3458mLR		gcaagcTTACTTTCAACCATAATAAATTCTTC (HindIII)
#3	3458mPF		gcaagctttaatAGTGAGGGCGCCAGGTTCG (HindIII)
#4	3458mPR		gcgaattCAACGCCTGTTCGCGCTGATGCTG (EcoRI)
#5	3459–61mLF	*PA3459* gene and *PA3459*–*61* operon deletion	gcggatccAGACGCTTGGAGTGGATTTC (BamHI)
#6	3459–61mLR		gcaagcttGCCGCACATATTCCTTACC(HindIII)
#7	3459mPF	*PA3459* gene deletion	gcaagctttaatagTGAAAATGGGCCCCCAACGC(HindIII)
#8	3459mPR		gcgaattcACAGGTCGCGGGCCAATTCC (EcoRI)
#9	3459–61mLF	*PA3459*–*61* operon deletion	gcaagctttagtaaTGAGCCCGCGCCGGCCT (HindIII)
#10	3459–61mLR		gcgaattcGTACTCGGCGTATGCGCCCAGG (EcoRI)
#11	3458eF	PA3458 expression	gcgaattcATGGTTGAAAGTAATAAGACCG (EcoRI)
#12	3458eR		gcgagctcCTGTCTTTCGTAGCGAAC (SacI)
#13	3458eF3		cgccatggATGGTTGAAAGTAATAAGACCGCTGC (NcoI)
#14	3458eR3		gcaagcttCTCTTCTTCGACGTCCTCTTCC (HindIII)
#15	3458eR4		gcggatccCTCTTCTTCGACGTCCTCTTCC (BamHI)
#16	3458pF	*PA3458* promoter cloning	cagaattcgcatgcCCAGGTCCATGATCTTCAGC (EcoRI/SphI)
#17	3458pR		gcggatccATAAATTCTTCGTTAAGGGTCCTGATAG (BamHI)
#18	3459pF	*PA3459* promoter cloning	cagaattcgcatgcCGTATCGGCTGAGACGCTTG (EcoRI/SphI)
#19	3459pR		gcggatccATTCCTTACCGGTTCTCCGTTG (BamHI)
#20	nadBqF	RT-qPCR	CTACCTGGACATCAGCCACA
#21	nadBqR		GGTAATGTCGATGCCGAAGT
#22	proCqF		CAGGCCGGGCAGTTGCTGTC
#23	proCqR		GGTCAGGCGCGAGGCTGTCT
#24	3458qF		TAATAAGACCGCTGCCGATACC
#25	3458qR		TGAGACGCTTGGAGTGGATTTC
#26	3459qF		CCCGGCTCAATGGGATGTTC
#27	3459qR		AGCGGTCGAGGCTGTAGTAG
#28	3973qF		GGATCCTGAAGTCGACGAGC
#29	3973qR		GAAAGCTGGAATGCGCCAC
#30	2204qF		CAGGTGGACTTCAGCATCCC
#31	2204qR		CGCCACTTGTTGAGGACTTC
#32	2252qF		GGGTGCCTACTTCACGATCC
#33	2252qR		ATCAGGGCCTGGAAGGAACT
#34	2264qF		CTACCAGCCTCGCTTCTTCA
#35	2264qR		CTGTCGGTCGATGAACTCCG
#36	2776qF		ACGGTATCCAATGCGACCTG
#37	2776qR		GGCGTCCATGATCTCCAACT
#38	3356qF		GGGCGAAACATCTTCAGCGA
#39	3356qR		GCTGGAAGGAGTTCACATGC
#40	3923qF		CTACTGCGAGAACGATGGCT
#41	3923qR		ACACTGGGCTTGAGGTTCAC
#42	4296qF		CTACCGTTGCGTAACCAGCA
#43	4296qR		GCTCTCGATCAGGCGGATAC
#44	4352qF		ATGAAACGAATCCTGGTGGC
#45	4352qR		CACGTTCAGCACGGTCAGTT
#46	5099qF		GACGTATAGCCGTAGTGGGC
#47	5099qR		GTGCTATCCGGACGATCCAC
#48	5100qF		GTGATCGTCTGCGACGGTAG
#49	5100qR		CAATCGATGGCGACCTGGTA
#50	5170qF		TCTTCTCCCTCCCGCAAAAC
#51	5170qR		AAGACGAAAGCGAGGGTGAG
#52	1270qF		CTATAGCGGCATGGTCCTGG
#53	1270qR		GATGCCTGGCCATAGAGTGC
#54	3461qF		CGGACCTCAACTACCTGCAA
#55	3461qR		CGCACGATGGTATCGGTGAA
